# Hungarian Linguistic, Cross-Cultural, and Age Adaptation of the Patient Satisfaction with Health Care in Inflammatory Bowel Disease Questionnaire (CACHE) and the Medication Adherence Report Scale (MARS)

**DOI:** 10.3390/children9081143

**Published:** 2022-07-29

**Authors:** Dóra Dohos, Alex Váradi, Nelli Farkas, Adrienn Erős, Andrea Párniczky, Eszter Schäfer, Éva Kosaras, Judit Czelecz, Péter Hegyi, Patrícia Sarlós

**Affiliations:** 1 Institute for Translational Medicine, Medical School, University of Pécs, 7624 Pécs, Hungary; dohos.dora@gmail.com (D.D.); varadi.alex09@gmail.com (A.V.); farkas.nelli@gmail.com (N.F.); andrea.parniczky@gmail.com (A.P.); hegyi2009@gmail.com (P.H.); 2 Szentágothai Research Centre, University of Pécs, 7624 Pécs, Hungary; 3 Heim Pál National Institute of Pediatrics, 1089 Budapest, Hungary; adriennhat@gmail.com; 4 Medical Centre, Hungarian Defence Forces, 1134 Budapest, Hungary; schafereszter@gmail.com; 5 Department of Pediatrics, Central Hospital of Borsod-Abaúj-Zemplén County and University Teaching Hospital, 3526 Miskolc, Hungary; kosaras.vica@gmail.com; 6 Bethesda Children’s Hospital, 1146 Budapest, Hungary; juditczelecz@yahoo.com; 7 Centre for Translational Medicine, Semmelweis University, 1085 Budapest, Hungary; 8 Heart and Vascular Center, Division of Pancreatic Diseases, Semmelweis University, 1122 Budapest, Hungary; 9 First Department of Medicine, Division of Gastroenterology, Medical School, University of Pécs, 7624 Pécs, Hungary

**Keywords:** adaptation, inflammatory bowel disease, TRANS-IBD, questionnaire, CACHE, MARS

## Abstract

Background: The TRANS-IBD study examines the superiority of joint transition visits, with drug adherence and patient satisfaction among the outcome measures. Our aim was a cross-cultural, age- and disease-specific adaptation of the ‘Medication Adherence Rating Scale’ (MARS) and ‘Patient satisfaction with health care in inflammatory bowel disease questionnaire’ (CACHE) questionnaires in patients with inflammatory bowel disease (IBD). Methods: Linguistic and cultural adaptation using test and re-test procedures were performed. Internal consistency with Cronbach’s α coefficients, confirmatory factor analyses with root Mean Square Error of Approximation (RMSEA), Comparative Fit Index (CFI), and Tucker-Lewis Index (TLI) were determined. Results: A total of 122 adolescents and 164 adults completed the questionnaires (47.5% male, mean age 17 ± 1; and 29.3% male, mean age 38 ± 11, respectively). In the MARS questionnaire, Cronbach’s α scores were found good in adolescents (0.864) and acceptable in adults (0.790), while in the CACHE questionnaire, scores were rated as excellent in both populations (0.906 and 0.945, respectively). The test-retest reliabilities were satisfactory in both groups (MARS questionnaire: r = 0.814 and r = 0.780, CACHE questionnaire: r = 0.892 and r = 0.898, respectively). RMSEA showed poor fit values in the MARS questionnaire and reasonable fit values in the CAHCE questionnaire, CFI and TLI had statistically acceptable results. Conclusion: Age-and disease-specific Hungarian versions of the questionnaires were developed, which are appropriate tools for TRANS-IBD RCT and daily IBD care.

## 1. Introduction

Inflammatory bowel disease (IBD) is becoming more common worldwide [1,2,3,4]. Approximately 25% of patients with IBD present before the age of 20 years. The annual incidence of pediatric and adult IBD in Hungary is 7.48 and 10.4 per 100,000 person-years, respectively [2,4]. In general, chronic illnesses have a significant impact on patients’ quality of life [5] and represent huge healthcare and economic burden [6,7].

In the case of a chronic illness diagnosed in childhood, multifaceted management should include appropriate infrastructure, treatment, continuing education, and collaboration between pediatricians and adult caregivers [8]. A good multidisciplinary team (MDT), including specialists and healthcare professionals, is essential in complex diseases such as cancer, heart disease, neurological rehabilitation and many gastrointestinal diseases to deliver high-quality care, optimize long-term outcomes and cost-benefit ratios. The primary goal of an MDT is to discuss treatment options and optimize them for the patient’s life, to ensure personalized therapy and appropriate compliance, thereby minimizing potential complications. MDT-driven care is now being introduced to IBD centres with standards that allow IBD-MDTs to be organized anywhere in the world. The IBD-MDT team should be composed of core (e.g., gastroenterologist, colorectal surgeon, IBD nurse, dietician, psychologist, radiologist) and extended members (e.g., histopathologist, dermatologist, rheumatologist). It is important to highlight that in the case of adolescents or young adult patients, pediatricians should also join the core members in order to make patient shift more efficient [9].

Patients may be shifted to the adult health care system with transfer or transition. Although transition is a dynamic, planned and disease-specific process, there has been much more positive feedback than after a single-step transfer [10,11]. The widely supported transition process is influenced not only by objective (e.g., appropriate management, financial difficulties) but also by subjective issues such as patient self-preparation, and differences in practice styles [12,13]. In addition to gaining patient cooperation, physicians should also seek parental support to improve adherence and reduce potentially negative health consequences [14].

However, the transition from pediatric to adult care is a crucial phase in patient management, but a method with strong evidence still does not exist [15,16]. To address this shortcoming, we designed a clinical trial called TRANS-IBD to assess the superiority of joint transition visits, which is considered the most supported method [15,16,17]. Based on international Delphi studies, the TRANS-IBD study takes into account individual, health care, and social outcomes [17,18,19,20]. Drug adherence is measured using the Medication Adherence Rating Scale (MARS) and patient satisfaction with the so-called CACHE questionnaire. The MARS questionnaire was validated by Horne et al. [21], in England for chronic diseases and has been adapted in various countries, such as Germany, Sweden and Portugal [22,23,24,25]. The CACHE questionnaire was validated by Casellas et al. [26], for the adult IBD population in Spain and has not been adapted yet but used in different countries, e.g., Canada, Italy and Spain [27,28,29]. Due to the increase in the number of research projects examining a common issue, such as transition, the need to adapt questionnaires for use outside the mother language is also increasing. A linguistically well-translated tool is far from enough; a cultural adaptation is also required to maintain its original conceptual and content validity. Cross-cultural adaptation is a unique method to use previously validated foreign questionnaires in different countries in different languages and to achieve comparable results [30].

We aimed to perform a cross-cultural, age- and disease-specific adaptation of the MARS and CACHE questionnaire, thus providing conceptually equivalent questionnaires with the original ones. Our primary target population was the younger generation of 16–19-years-old with IBD to obtain the tools needed for the TRANS-IBD study. Furthermore, the adult population was also included so that the tool could later be used in a wider range of patients with IBD.

## 2. Materials and Methods

The study protocol confirms the ethical guidelines of the Declaration of Helsinki, updated in 2013, as reflected in a prior approval by the institution’s human research committee. The ethical approval of the TRANS-IBD study, extended by this cross-sectional multicentric survey, was received from the Scientific and Research Ethics Committee of the Medical Research Council (IV/3260-1/2021/EKU). All patients involved and their parents/legal guardians provided their written informed consent to their participation in the study and the anonymous data analysis.

### 2.1. Patient Sample and Data Collection

The adaptation of the questionnaires was performed in adolescents with IBD aged 15–19-years between March 2020 and June 2021. The paper-based surveys were collected in nine Hungarian hospitals and later uploaded to our electronic database. Later, the adaptation process was extended and ran on an online platform for adults with IBD. A data sheet was also collected on which several personal and disease-related parameters were recorded, ethnicity was distinguished as a representative of the Hungarian ethnic group and the ethnic minority.

### 2.2. Characteristics of the Adapted Questionnaires: MARS and CACHE

The MARS questionnaire is a non-disease, non-transition specific tool for assessing medication adherence. The original questionnaire, validated by Horne et al., consisted of five items for which patients had to choose one of the five responses: never, rarely, sometimes, often, always corresponding to 1–5 points. The total score ranged from 5 to 25. Higher scores indicate better adherence to the recommended medication [21]. In the Italian adaptation, five categories were used to distinguish patient adherence, as follows: ‘never adherent’ (scores 5–9); ‘seldom adherent’ (scores 10–14); ‘sometimes adherent’ (scores 15–19); ‘often adherent’ (scores 20–24); ‘always adherent’ (score 25) [25]. The application for the questionnaire adaptation permit has been sent to the authors several times since January 2020, but no response was received until the completion of our adaptation process.

The CACHE questionnaire is a non-transition specific tool for measuring the subjective opinion of patients with IBD about the quality of care. The original tool, validated by Casellas et al., consisted of 31 items in six structured domains: staff care (10 items); clinical care (5 items); centre facilities (4 items); patient information (5 items); accessibility (4 items) and patient support (3 items). Patients should choose from the following five options to answer: ‘Totally agree’; ‘Agree’; ‘Neither agree nor disagree’; ‘Disagree’; and ‘Totally disagree’. The final score was standardized to range from 0 (lowest level of satisfaction) to 100 (maximum satisfaction). The scores were standardized using the formula applied to the score for each individual item: (real score-minimum score)/(maximum score-minimum score) × 100 [26]. The authorization for the adaptation process and the use of the questionnaire was approved by Casellas via e-mail in December 2019.

### 2.3. Cross-Cultural Adaptation

Linguistic adaptation through translation and cultural adaptation using test and re-test procedures were led by the guideline of Beaton et al. (Supplementary Figure S1) [30]. First, two independent translators prepare questionnaire forms in the target Hungarian language (T1, T2), then D.D., P.S. and A.E. compared copies of the two translations and evaluated grammatic or conceptual discrepancies and synthesized them (T1,2). Next, T1,2 was translated back to the original English language by two other translators (BT1, BT2). Then D.D., P.S., A.E., language professionals and translators compared the back-translated versions to the original survey and tried to find any semantic differences that may appear in the T1,2 due to forward and backward translational processes. In the fifth phase, we performed a pre-test involving 30 patients from our target population. In the final step, we involved eight Hungarian hospitals to test and re-test the questionnaires with adolescents, and online surveys were available for adults. At least four-five individuals per question were selected to complete the survey, and the same questionnaire as re-testing was repeated within seven days. The second questionnaires were returned in a stamped envelope, while reminder e-mail was sent for the online surveys.

### 2.4. Statistical Analyses

Demographic data were summarized using descriptive statistics. Mean, standard deviation (SD), median, first and third quartiles, and minimum and maximum values were given for continuous variables. Event numbers and percentages were calculated for categorical data. In case of incomplete participation, the unanswered items of the CACHE questionnaire were filled in with the median values of the available answers.

A weighted least squared method (WLSMV) estimator was used since it is recommended as a good alternative if the data are non-normal due to the ordinal nature of the scale (e.g., the Likert scale is less than seven points) [31]. Confirmatory factor analysis was conducted to assess the fit of the original model, root means square error of approximation (RMSEA), comparative fit index (CFI), Tucker-Lewis index (TLI), and scaled Chi-Square were used. A cut-off criterion of 0.90 or higher has been recommended for CFI and TLI. In addition, RMSEA values less than 0.08 are considered acceptable [32].

The floor and ceiling effect was determined when more than 15% of the patients marked extreme values.

Reliability refers to the consistency of a measure. The reliability across items, i.e., internal consistency was determined using Cronbach’s α coefficients. Statistically, α = 0.70 is the minimum acceptable value, while ≥0.9 can be interpreted as excellent internal consistency. The test-retest reliability was evaluated by Spearman’s rank correlation of the total scores of the questionnaire, which is statistically acceptable ≥0.7, however, the reliability also depends on the expected stability of the construct to be measured.

The relationship between demographic variables and questionnaire totals or subscores was tested by Spearman’s rank correlation for continuous variables, Mann-Whitney U-test or Kruskal-Wallis rank sum test for categorical variables.

The results were considered significant if *p* < 0.05. All statistical analysis were conducted using the R programming language (R Core Team, 2021, Vienna, Austria, R version 4.1) and the lavaan R package [33,34].

## 3. Results

### 3.1. Characteristics of Patients Involved in the Adaptation Process

A total of 122 adolescents with IBD participated from nine different IBD centres in Hungary. The median number of patients enrolled in the various centres was 10 (range 4–34). Of the 122 adolescents involved, 58 were male and 65 were female; 80 patients had Crohn’s disease (CD) and 42 had ulcerative colitis (UC). The mean age was 17.21 ± 0.95, and the mean disease duration time was 4 ± 2.92 years. The majority of patients attended secondary education (51% in high school) (Table 1).

Out of 70 different cities in Hungary, 164 adults with IBD participated in the adaptation process. 45 of them were male and 119 were female; 100 had CD and 67 UC. The mean age was 37.80 ± 12.67 years and the mean disease duration was 10 ± 8.07 years. The majority of volunteers (66%) were employed (Table 1).

### 3.2. MARS

#### 3.2.1. Questionnaire Performance

The questionnaire was well understood during the pre-test process, no additional changes to the phrases were required (Supplementary Table S1). Due to the shortness of the survey, it could be completed in a short time, so the proportion of unfilled surveys was 4.10% for adolescents and 0.60% for adults (Table 2 and Table 3).

However, the floor effect was not, but the ceiling effect was observed in all items in both patient groups. The most pronounced ceiling effect was detected in question 2 and 5, where more than 80% of the participants in the groups answered ‘Never’, separately.

#### 3.2.2. Questionnaire Adequacy

Confirmatory factor analysis was used to evaluate the construct validity, to verify a given theory-based factor structure throughout RMSEA, CFI, TLI. RMSEA showed poor fit values, CFI and TLI had statistically acceptable results in both adolescent and adult populations (RMSEA: 0.174 [CI: 0.105-0.250] and 0.232 [CI: 0.112-0.232]; CFI: 0.992 and 0.991; TLI: 0.985 and 0.982, respectively) (Table 4).

#### 3.2.3. Reliability

The value of internal consistency was good in adolescents and acceptable in adults (Cronbach’s α: 0.864 and 0.790, respectively). In the adolescent population, 42% of patients completed and returned retest copies, while 46% of adults sent back them on time. The test-retest reliability was satisfactory in both groups, with good (*r* = 0.814; *p* = 0.001) and acceptable (*r* = 0.780; *p* = 0.001) results (Table 3).

#### 3.2.4. Influence of Patient Demographics on Drug Adherence

Among adolescents, patients without previous surgery achieved significantly higher overall scores than patients who underwent surgery (24 ± 2.18 vs. 21 ± 5.10, respectively; *p* = 0.034) (Supplementary Table S2). In the adult population, none of the analysed demographic parameters showed a significant correlation with the total score (Supplementary Table S2).

#### 3.2.5. Adherence of Hungarian Patients with IBD

Based on the categories created in the Italian adaptation, a significant part of the Hungarian patients was qualified as ‘often’ or ‘always’ adherent (58.1% and 33.3% in the adolescent group, 54.6% and 35.0% in the adult group, respectively). Overall scores were high in both populations, the descriptive values of the subscores are detailed in Supplementary Table S3.

### 3.3. CACHE

#### 3.3.1. Questionnaire Performance

During the pre-test process minimal changes in the phrases were needed (Supplementary Table S4). The survey was relatively long, so it took more time to complete it, but the rate of blank questionnaires was 0% in both populations (Table 5 and Table 6).

In the adolescent population, the floor effect was observed in question 11, and the ceiling effect was reached in all questions except questions 11 and 14. The most pronounced ceiling effect was observed in question 2, where 76% of participants answered, ‘Totally agree’. In the adult population, the floor effect was reached only for question 14, and the ceiling effect for all items except question 11. The most pronounced ceiling effect occurred in the same item as in the young population with 63% of responses. This question was about whether the patient trusted the doctor’s judgment regarding treatment.

#### 3.3.2. Questionnaire Adequacy

RMSEA showed reasonable fit values, CFI and TLI showed statistically acceptable results in the adaptation studies in both adolescent and adult populations (RMSEA: 0.074 [CI: 0.060–0.081] and 0.074 [CI: 0.055–0.072]; CFI: 0.988 and 0.998; TLI: 0.987 and 0.998, respectively) (Table 3).

#### 3.3.3. Reliability

The value of internal consistency was excellent in both populations (Cronbach’s α: 0.906 and 0.945, respectively) (Table 3). The analysis was also performed for the 6 domains separately, where Cronbach’s α ranged from 0.513 to 0.872 in adolescents and from 0.415 to 0.919 in the adult population (Supplementary Table S5). 42% of the adolescents completed and transferred the retest tools, while 46% of adults returned the second questionnaire promptly. Test-retest reliability was satisfactory in both studies (*r* = 0.892; *p* = 0.001 and *r* = 0.898; *p* = 0.001, respectively) (Table 3).

#### 3.3.4. The Effect of Patient Demographics on Satisfaction with the Quality of Care

Among the adolescents, a significant difference between demographic parameters was measured only in the domain measuring staff care. The Hungarian ethnic group scored significantly higher than the other ethnic minority group (83 ± 15 vs. 71 ± 12, respectively; *p* = 0.030). Significantly lower subscores were observed in patients with CD than in patients with UC (80 ± 15 vs. 86 ± 14, respectively; *p* = 0.019) (Supplementary Table S6).

In the adult population, significantly higher scores were found in overall scores of men than in women (77 ± 13 vs. 70 ± 17; *p* = 0.019), and in subscores of men (82 ± 15 vs. 74 ± 20; *p* = 0.027 in the staff care domain, 86 ± 14 vs. 74 ± 20; *p* < 0.001 in the clinical care items, 65 ± 18 vs. 56 ± 22; *p* = 0.027 in the patient information items, respectively). According to the clinical care domain, the more medication patients took, the more satisfied they were (70 ± 20 in patients without medication vs. 82 ± 16 in patients with 1–2 medications vs. 94 ± 10 in patients with 3–4 medications, respectively; *p* = 0.002). The duration of the disease also had a positive effect on satisfaction with clinical care (ρ: 0.190, *p* = 0.016). Satisfaction was higher in patients with CD compared to patients with UC in the overall scores (75 ± 14 vs. 67 ± 18; *p* = 0.014) and in the domains of staff care (79 ± 16 vs. 71 ± 22; *p* = 0.042), clinical care (85 ± 14 vs. 78 ± 18; *p* = 0.017) and accessibility (74 ± 19 vs. 64 ± 24; *p* = 0.010), respectively. Patients receiving biological therapy were overall more satisfied than patients without biological treatment (77 ± 13 vs. 69 ± 17; *p* = 0.005) and in the domains measuring staff care (82 ± 15 vs. 72 ± 20; *p* = 0.006), clinical care (87 ± 13 vs. 79 ± 17; *p* = 0.006), centre facilities (84 ± 13 vs. 73 ± 21; *p* = 0.003), accessibility (75 ± 22 vs. 68 ± 21; *p* = 0.032), and patient support (67 ± 19 vs. 57 ± 20; *p* = 0.001), respectively (Supplementary Table S7).

#### 3.3.5. Satisfaction with the Quality of Care among Hungarian Patients with IBD

The overall score of the questionnaire was above 70 points in both the adolescents and adult groups, representing a relatively good satisfaction with the health care system. The lowest mean scores were observed in domains 4 and 6, which evaluated patient information and support. The mean scores were nearly the same in the groups, with only a larger difference in question 26, which analysed communication between the medical team and other health care workers (Supplementary Table S8).

## 4. Discussion

The MARS questionnaire was well-accepted by the participants. Self-reported medication adherence rating scales can be highly biased due to direct and indirect influencing factors, e.g., hospital environment, distrust of the doctor, and self-criticism. This possible bias was detected when analysing the floor-ceiling effect values; zero and low floor effects were observed, but ceiling effect appeared in all 5 items. The highest ceiling effect was observed in both groups in items 2 and 5, where more than 80% of the patients stated that prescribed dose and amount of the medication had never changed.

The questionnaire adequacy results were considered acceptable values in both populations. The CFI and TLI indices indicate relatively good model–data fit outcomes, however the RMSEA values showed a poor fit. Reliability scores showed good and acceptable internal consistency, since overall result in the group of adolescents and adults exceeds the limit proposed for group comparison. Furthermore, the test-retest reliability with Pearson correlation was rated as good and acceptable in both groups analysed. However, our *r*-value was higher than in other studies [22,24,25] but lower than that of Bäck et al. [23]. The discrepancy between the values may appear due to altered intervals between the completion of the questionnaires, which result in a real change in adherence rather than a low inconsistency quality [22]. However traditionally decreased drug-taking compliance is assumed, but our data showed that medication adherence was relatively high in Hungary.

The distribution of mean scores was distorted, however our results were in line with those of Mahler et al. [22]. Based on the mean scores, the majority of patients showed appropriate adherence. According to the literature, self-reported questionnaires overestimate their outcomes; thus, this type of measurement is more powerful in identifying subjects’ deficiencies and non-adherence than their positive sides [22,35,36].

So far, after validation of the CACHE questionnaire by Casellas et al. [26], the survey has been used in different countries [27,28,29], but foreign adaptation has not yet been published. According to our participants, the questionnaire was relatively long, however, all patients answered the questions. Patients with chronic illnesses go more often than average in hospital, so it was interesting to evaluate the floor-ceiling effect, and to see how satisfied our patients are with the Hungarian health care service. The ceiling effect was not reached in the case of item 11 (opinion about the price of drugs), which can be explained by the state-funded patient care and drug subsidies in Hungary [37]. The observed high satisfaction and ceiling effects may be due to their specific over-controlled situation, e.g., more frequent doctor-patient visits, more consultation times. Our results did not differ from the conclusion observed in the study of Casellas et al., in 35 Spanish hospitals [29].

RMSEA, CFI and TLI, were considered acceptable in both populations. In addition, the relatively good model-data fit outcomes were indicated by CFI and TLI, the RMSEA results. The overall reliability scores, the overall Cronbach’s α coefficient showed excellent internal consistency results. The scores of internal consistencies for the six dimensions did not reach the recommended minimum in all cases. In the adolescent group, domains 1 and 2, and in adults, domains 1, 2, 3 and 5 should be used separately for comparisons, but this is not acceptable for the remaining domains, they can be used only in their entirety. The overall and subdomain results in the adult population showed the same or nearly the same Cronbach’s α coefficient as in the original study by Casellas et al. [26]. Furthermore, the test-retest reliability was rated as good in both groups.

Assessing patient satisfaction with health care can help identify possible strengths, weaknesses and areas for improvement in local health services, and monitor improvements in the quality of care [26]. Adolescents had higher total scores and subscores compared to adults, except for nearly identical results in the patient support measuring domain. Assessing staff care and accessibility, significantly higher overall scores and subscores were detected in patients with CD compared to patients with UC and in patients on biological therapy than in patients without biological therapy. Patients receiving biological therapy also scored significantly higher in terms of centre facilities and patient support than those not receiving biologics. As previously expected, those with CD or long-term illness or those taking multiple medications or biologic therapies scored significantly higher in the clinical care setting.

In contrast to the validation study, our mean subscores were slightly lower in both populations. Nevertheless, the mean overall scores were over 70, indicating relatively acceptable and in all cases improving patient satisfaction.

Our study has several strengths and limitations. During the adaptation process, a rigorous methodology was followed. Patients from different types of centres and many different regions in Hungary were involved ensuring that the findings can be generalized. Our study suffers from limitations, as well. Due to the pandemic situation in 2020 and 2021, we lost some centres, thus the number of participants was lower than expected. The adaptation process of the adult population run via the internet, so personal contact was not possible.

In summary, we successfully completed a cross-cultural, age- and disease-specific adaptation of two easy-to-use, self-report questionnaires to measure medication adherence and patient satisfaction with health care in Hungary for adolescents and adults with IBD. Based on our results, these Hungarian questionnaires are feasible, reliable, reproducible, and comparable with the original validated versions. We believe that these questionnaires can help clinicians obtain information not only about medication adherence, but also about patient satisfaction, which may facilitate the effectiveness and utility of MDTs. MDT-driven care is already successfully applied in IBD centres in Europe, and we hope that with these adapted questionnaires, existing and future Hungarian MDTs will have more tools for patient-centred management and quality progress [38,39].

## Figures and Tables

**Table 1:** Main characteristics of the adolescents and adult patients involved.

*Characteristics*	*Adolescent* *Population (n = 122)*	*Adult* *Population (n = 164)*	*Overall (n = 286)*
*male/female*	58/64	45/119	103/183
*ethnicity: Hungarian/other*	115/6	156/8	271/14
*age (mean ± SD; yrs)*	17 ± 1	38 ± 11	29 ± 13
*disease duration time (mean ± SD; yrs)*	10 ± 8	4 ± 3	7 ± 7
*Crohn’s disease/ ulcerative colitis*	80/42	100/64	180/106
*previous intestinal surgery (%)*	17	35	27
*comorbidities (%)*	15	34	26
*therapy (%): biologicals*	44	39	41
*steroids*	25	18	21
*azathioprine*	37	39	38
*5-ASA*	59	56	55

*n*: number; SD: standard deviation; yrs: years; 5-ASA: 5-aminosalicylate.

**Table 2:** Distribution of adolescent responses in MARS questionnaire.

*Questions*	*Always*	*Often*	*Occasionally*	*Rarely*	*Never*
*I forget to take the medicine.*	1 (1%)	6 (5%)	21 (18%)	44 (38%)	45 (38%)
*I alter the dose of medicine.*	1 (1%)	1 (1%)	3 (3%)	7 (5%)	105 (90%)
*I stop taking the medicine for a while.*	1 (1%)	2 (2%)	4 (3%)	9 (8%)	101 (86%)
*I decided to miss out a dose.*	1 (1%)	2 (2%)	7 (5%)	9 (8%)	98 (84%)
*I take less than instructed.*	1 (1%)	1 (1%)	4 (3%)	6 (5%)	105 (90%)

**Table 3:** Distribution of adult responses in MARS questionnaire.

*Questions*	*Always*	*Often*	*Occasionally*	*Rarely*	*Never*
*I forget to take the medicine.*	1 (1%)	9 (5%)	23 (14%)	58 (36%)	72 (44%)
*I alter the dose of medicine.*	0 (0%)	3 (2%)	6 (4%)	19 (12%)	135 (82%)
*I stop taking the medicine for a while.*	1 (1%)	7 (4%)	10 (6%)	25 (15%)	120 (74%)
*I decided to miss out a dose.*	0 (0%)	3 (2%)	11 (7%)	27 (16%)	122 (75%)
*I take less than instructed.*	1 (1%)	2 (1%)	6 (4%)	17 (10%)	137 (84%)

**Table 4:** Adequacy and reliability indices of the MARS (A) and CACHE (B) questionnaire in the two adaptation groups.

Indices	MARS Questionnaire		CACHE Questionnaire	
	Adolescent Population	Adult Population	Adolescent Population	Adult Population
number of responses (*n*)	117	163	122	164
total score, mean (SD)	23 (2.907)	23 (2.756)	76 (12.369)	72 (16.235)
CFI	0.987	0.971	0.937	0.971
TLI	0.973	0.942	0.930	0.968
RMSEA (CI)	0.174 (0.105–0.250)	0.169 (0.112–0.232)	0.071 (0.060–0.081)	0.063 (0.055–0.72)
Cronbach’s α	0.864	0.790	0.906	0.945
number or retests (*n*)	51	76	52	76
total score, mean (SD)	24 (2.880)	24 (2.154)	70 (16.276)	72 (17.551)
test-retest: ρ (p)	0.814 (0.001)	0.780 (0.001)	0.892 (0.001)	0.898 (0.001)

*n*: number; SD: standard deviation; CFI: comparative fit index, TLI: Tucker-Lewis index, CI: confidence interval.

**Table 5:** Distribution of adolescent responses to CACHE questionnaire.

*Questions*	*Totally Agree*	*Agree*	*Occasionally*	*Neither Agree nor* *Disagree*	*Disagree*
*My doctor spends an appropriate amount of time listening to and answering my questions about my bowel disease*	84 (69%)	29 (24%)	8 (6%)	1 (1%)	0 (0%)
*I have confidence in my doctor’s judgment when managing and treating my bowel disease*	93 (76%)	18 (15%)	10 (8%)	1 (1%)	0 (0%)
*understand the explanations given to me on my bowel disease, its treatment, and the side effects of treatment*	62 (51%)	49 (40%)	10 (8%)	0 (0%)	1 (1%)
*I get advice and guidance about nutrition, daily activities, exercise, etc, which I have to follow because of my bowel disease*	55 (45%)	36 (30%)	21 (17%)	10 (8%)	0 (0%)
*My doctor takes my opinion and preferences regarding treatment for my bowel disease into account*	64 (52%)	44 (36%)	11 (9%)	2 (2%)	1 (1%)
*The medical personnel who look after me know my medical history and concern themselves with the evolution of my bowel disease*	65 (53%)	40 (33%)	13 (11%)	3 (2%)	1 (1%)
*The center I go to have my condition treated is well-located and easily accessible*	52 (43%)	40 (33%)	18 (15%)	9 (7%)	3 (2%)
*The facilities at the hospital I go to treat my bowel disease are adequate and comfortable*	57 (46%)	43 (35%)	18 (15%)	2 (2%)	2 (2%)
*Communication with the medical staff treating me is appropriate and fluid*	57 (46%)	43 (35%)	18 (15%)	2 (2%)	2 (2%)
*I feel listened to and understood by the medical staff treating me when I explain my intestinal problems and the difficulties they cause me*	66 (54%)	42 (34%)	13 (11%)	0 (0%)	1 (1%)
*I worry about the price I have to pay for the drugs prescribed for my bowel disease*	9 (7%)	15 (12%)	22 (18%)	36 (30%)	40 (33%)
*For me, it is important that I always see the same medical team*	31 (26%)	42 (34%)	37 (30%)	10 (8%)	2 (2%)
*The staff take into account the consequences of my bowel disease treatment on my daily life*	61 (50%)	48 (39%)	12 (10%)	1 (1%)	0 (0%)
*I have been informed about how to contact with patients’ associations for people with intestinal problems like mine*	20 (16%)	33 (27%)	24 (20%)	24 (20%)	21 (17%)
*Having a specialist nurse in the medical team treating me would help me with my bowel disease*	28 (23%)	35 (29%)	38 (31%)	12 (10%)	9 (7%)
*The staff that look after me and the place I go for treatment motivate me to stick with the treatment for my illness*	62 (51%)	41 (33%)	12 (10%)	5 (4%)	2 (2%)
*The center where they administer my medication has the necessary resources and facilities*	73 (60%)	37 (30%)	9 (7%)	2 (2%)	1 (1%)
*At the hospital where I get treatment for my bowel disease, I can get information about my disease through brochures, information campaigns, etc*	24 (20%)	36 (30%)	37 (30%)	14 (11%)	11 (9%)
*I can see the clinician when I have a flare-up*	84 (69%)	27 (22%)	4 (3%)	7 (6%)	0 (0%)
*Being able to talk with people who have the same or similar problems as me while I am receiving my medication, helps me to share questions and concerns related to my bowel disease*	21 (17%)	27 (22%)	32 (27%)	28 (23%)	13 (11%)
*Visits can be scheduled on days and at times that least affect my daily activities (work, studies…)*	39 (32%)	37 (30%)	24 (20%)	15 (12%)	7 (6%)
*In the hospital, they treat me with sufficient intimacy and reserve*	76 (63%)	36 (29%)	5 (4%)	3 (2%)	2 (2%)
*The time I have to wait before being seen at the visit is reasonable*	43 (35%)	46 (38%)	23 (19%)	7 (6%)	3 (2%)
*In the center I go to for treatment, I can be attended over the phone*	68 (56%)	34 (28%)	17 (14%)	3 (2%)	0 (0%)
*The bathrooms in the center are adequate and accessible*	36 (30%)	44 (36%)	27 (22%)	9 (7%)	6 (5%)
*There is good coordination and communication between my medical team and other specialists and/or primary care*	46 (38%)	38 (31%)	23 (19%)	12 (10%)	3 (2%)
*I’m satisfied with the results of the treatment I receive*	58 (48%)	38 (31%)	22 (18%)	4 (3%)	0 (0%)
*If any problems arise with the treatment I am receiving, my medical team resolve it quickly and effectively*	63 (52%)	47 (39%)	8 (6%)	4 (3%)	0 (0%)
*I feel safer if I get the treatment at the hospital than if I had to do it at home*	38 (31%)	25 (20%)	32 (27%)	17 (14%)	10 (8%)
*I understand the instructions I’ve been given about my medication*	73 (60%)	34 (27%)	12 (10%)	1 (1%)	2 (2%)
*I’ve been given adequate information about the side effects of my medication*	61 (50%)	38 (31%)	17 (14%)	4 (3%)	2 (2%)

**Table 6:** Distribution of adult responses in CACHE questionnaire.

*Questions*	*Totally Agree*	*Agree*	*Occasionally*	*Neither Agree nor* *Disagree*	*Disagree*
*My doctor spends an appropriate amount of time listening to and answering my questions about my bowel disease*	89 (54%)	36 (22%)	31 (19%)	7 (4%)	1 (1%)
*I have confidence in my doctor’s judgment when managing and treating my bowel disease*	104 (63%)	34 (21%)	24 (15%)	1 (1%)	1 (1%)
*understand the explanations given to me on my bowel disease, its treatment, and the side effects of treatment*	79 (48%)	63 (39%)	20 (12%)	2 (1%)	0 (0%)
*I get advice and guidance about nutrition, daily activities, exercise, etc, which I have to follow because of my bowel disease*	58 (35%)	41 (25%)	44 (27%)	13 (8%)	8 (5%)
*My doctor takes my opinion and preferences regarding treatment for my bowel disease into account*	77 (47%)	51 (31%)	28 (17%)	7 (4%)	1 (1%)
*The medical personnel who look after me know my medical history and concern themselves with the evolution of my bowel disease*	75 (46%)	42 (25%)	28 (17%)	13 (8%)	6 (4%)
*The center I go to have my condition treated is well-located and easily accessible*	87 (53%)	51 (31%)	22 (14%)	2 (1%)	2 (1%)
*The facilities at the hospital I go to treat my bowel disease are adequate and comfortable*	76 (46%)	53 (32%)	26 (16%)	6 (4%)	3 (2%)
*Communication with the medical staff treating me is appropriate and fluid*	80 (49%)	39 (24%)	32 (19%)	9 (6%)	4 (2%)
*I feel listened to and understood by the medical staff treating me when I explain my intestinal problems and the difficulties they cause me*	74 (45%)	60 (37%)	20 (12%)	8 (5%)	2 (1%)
*I worry about the price I have to pay for the drugs prescribed for my bowel disease*	17 (11%)	18 (11%)	48 (29%)	46 (28%)	35 (21%)
*For me, it is important that I always see the same medical team*	70 (42%)	64 (39%)	23 (14%)	6 (4%)	1 (1%)
*The staff take into account the consequences of my bowel disease treatment on my daily life*	63 (39%)	67 (40%)	23 (14%)	10 (6%)	1 (1%)
*I have been informed about how to contact with patients’ associations for people with intestinal problems like mine*	32 (19%)	46 (28%)	25 (16%)	29 (18%)	32 (19%)
*Having a specialist nurse in the medical team treating me would help me with my bowel disease*	32 (19%)	67 (40%)	37 (24%)	20 (12%)	8 (5%)
*The staff that look after me and the place I go for treatment motivate me to stick with the treatment for my illness*	72 (44%)	64 (39%)	20 (12%)	6 (4%)	2 (1%)
*The center where they administer my medication has the necessary resources and facilities*	86 (52%)	48 (29%)	24 (15%)	5 (3%)	1 (1%)
*At the hospital where I get treatment for my bowel disease, I can get information about my disease through brochures, information campaigns, etc*	47 (28%)	41 (25%)	34 (21%)	26 (16%)	16 (10%)
*I can see the clinician when I have a flare-up*	73 (45%)	53 (32%)	24 (15%)	10 (6%)	4 (2%)
*Being able to talk with people who have the same or similar problems as me while I am receiving my medication, helps me to share questions and concerns related to my bowel disease*	31 (19%)	40 (24%)	40 (24%)	32 (19%)	21 (14%)
*Visits can be scheduled on days and at times that least affect my daily activities (work, studies…)*	53 (32%)	42 (25%)	30 (18%)	25 (16%)	14 (9%)
*In the hospital, they treat me with sufficient intimacy and reserve*	73 (45%)	59 (36%)	20 (12%)	8 (5%)	4 (2%)
*The time I have to wait before being seen at the visit is reasonable*	40 (25%)	59 (36%)	41 (25%)	20 (12%)	4 (2%)
*In the center I go to for treatment, I can be attended over the phone*	63 (39%)	51 (31%)	28 (17%)	15 (9%)	7 (4%)
*The bathrooms in the center are adequate and accessible*	42 (25%)	51 (31%)	42 (25%)	13 (8%)	16 (11%)
*There is good coordination and communication between my medical team and other specialists and/or primary care*	36 (22%)	40 (24%)	39 (24%)	29 (18%)	20 (12%)
*I’m satisfied with the results of the treatment I receive*	60 (37%)	54 (33%)	38 (23%)	8 (5%)	4 (2%)
*If any problems arise with the treatment I am receiving, my medical team resolve it quickly and effectively*	65 (40%)	55 (33%)	31 (19%)	9 (6%)	4 (2%)
*I feel safer if I get the treatment at the hospital than if I had to do it at home*	41 (25%)	48 (29%)	48 (29%)	20 (12%)	7 (5%)
*I understand the instructions I’ve been given about my medication*	99 (60%)	56 (34%)	8 (5%)	1 (1%)	0 (0%)
*I’ve been given adequate information about the side effects of my medication*	57 (35%)	48 (29%)	35 (21%)	13 (8%)	11 (7%)

## Data Availability

Data is contained within the article or supplementary material. The data presented in this study are available in the article and its supplementary material.

## Supplementary Materials

The following supporting information can be downloaded at: https://www.mdpi.com/article/10.3390/children9081143/s1, Figure S1: Steps of adaptation; Table S1: The Hungarian version of the MARS questionnaire; Table S2: Correlation between the total scores of MARS and demographic data; Table S3: Mean scores from the MARS questionnaire for adolescents and adults; Table S4: The Hungarian version of the CACHE questionnaire; Table S5: The mean and the Cronbach’s α values in the CACHE questionnaires; Table S6: Correlation between total and subscores of the CACHE questionnaire and demographic data in the adolescent population; Table S7: Correlation between total and subscores of the CACHE questionnaires and demographic data in the adult population; Table S8: Mean scores from the CACHE questionnaires for adolescents and adults.

## Abbreviations

CACHE	Patient satisfaction with health care in inflammatory bowel disease questionnaire
CD	Crohn’s disease
CFI	comparative fit index
IBD	inflammatory bowel disease
MARS	Medication adherence report scale
MDT	multi-disciplinary team
RMSEA	root mean square error of approximation
TLI	Tucker-Lewis index
UC	ulcerative colitis
