# Supplementary material for: Hungarian Linguistic, Cross-Cultural, and Age Adaptation of the Patient Satisfaction with Health Care in Inflammatory Bowel Disease Questionnaire (CACHE) and the Medication Adherence Report Scale (MARS)
Source: Children (Basel). 2022 Jul 29;9(8):1143. doi: 10.3390/children9081143 (PMC9406584; doi:10.3390/children9081143)

Supplementary Table S1 The Hungarian version of the MARS questionnaire

Medication Adherence Report Scale (MARS)<br>**Gyógyszerszedési szokásokat felmérő skála**

Please mark the square in each row which best represents your habits of taking medication in the past 1 week. There is no right or wrong answer.

Kérjük, jelöld be mindegyik sorban azt az egy négyzetet, amely a legjobban jellemzi a gyógyszerszedési szokásaidat **az elmúlt egy hétben**. Nincs helyes vagy rossz válasz.

**Original version**	**Hungarian version**
1. I forget to take the medicine.	1. Elfelejtem bevenni a gyógyszert.
2. I alter the dose of medicine.	2. Megváltoztatom a gyógyszer adagját.
3. I stop taking the medicine for a while.	3. Egy időre abbahagyom a gyógyszer szedését.
4. I decided to miss out a dose.	4. Úgy döntöttem, kihagyok egy gyógyszer adagot.
5. I take less than instructed.	5. Kevesebbet veszek be, mint ami elő van írva.

Supplementary Table S2 Correlation between the total scores of MARS and demographic data

demographic variables	***total score of MARS (p)***	
	**adolescent population**	**adult population**
gender	0.171	0.375
ethnicity	0.865	0.504
age at adaptation[1]	0.880	0.859
disease duration time[1]	0.213	0.653
disease type	0.601	0.236
previous intestinal surgery	**0.034**	0.944
biological treatment	0.490	0.258
steroid treatment	0.572	0.201
azathioprine treatment	0.708	0.687
5-ASA treatment	0.831	0.583
comorbidities	0.701	0.924

[1]: results are given in correlation coefficients (ϱ)

Significant results shown in bold.

Supplementary Table S3 **Mean scores from the MARS questionnaire for adolescents and adults**

	Scores	Mean scores (SD)	
		adolescent population	adult population
1	I forget to take the medicine	4.1 (±0.9)	4.2 (±0.9)
2	I alter the dose of medicine.	4.8 (±0.6)	4.8 (±0.6)
3	I stop taking the medicine for a while.	4.8 (±0.7)	4.6 (±0.8)
4	I decided to miss out a dose.	4.7 (±0.7)	4.6 (±0.7)
5	I take less than instructed.	4.8 (±0.6)	4.8 (±0.6)
	Total score	23.2 (±2.9)	22.9 (±2.8)

Supplementary Table S4 The Hungarian version of the CACHE questionnaire

The IBD CACHE questionnaire
Az egészségügyi ellátással kapcsolatos betegelégedettségi kérdőív (CACHE)

Please select one box in each row which is most characteristic of you with respect to your satisfaction! There is no right or wrong answer.

Kérjük, jelöld be mindegyik sorban azt az egy négyzetet, amely a legjobban jellemző az elégedettségeddel kapcsolatban!
Nincs helyes vagy rossz válasz.

Original version	Hungarian version
1. My doctor spends an appropriate amount of time listening to and answering my questions about my bowel disease	1. Az orvosom megfelelő mennyiségű időt szán arra, hogy meghallgassa és megválaszolja a betegségemmel kapcsolatos kérdéseimet.
2. I have confidence in my doctor's judgment when managing and treating my bowel disease	2. Megbízom az orvosom gyógyításom és kezelésem során hozott döntéseiben.
3. I understand the explanations given to me on my bowel disease, its treatment, and the side effects of treatment	3. Megértem a gyulladásos bélbetegségemmel, a kezelésemmel és a gyógyszerek mellékhatásaival kapcsolatos magyarázatokat.
4. I get advice and guidance about nutrition, daily activities, exercise, etc, which I have to follow because of my bowel disease	4. Tanácsokat és útmutatást kapok a táplálkozásommal, a napi tevékenységemmel kapcsolatban, amiket követnem kell a betegségem miatt.
5. My doctor takes my opinion and preferences regarding treatment for my bowel disease into account	5. A kezelésem során az orvosom figyelembe veszi a bélbetegségem kezelésével kapcsolatos véleményem és azt, hogy mit részesítek előnyben.
6. The medical personnel who look after me know my medical history and concern themselves with the evolution of my bowel disease	6. A gondozásomban résztvevő egészségügyi személyzet ismeri a betegségtörténetemet és foglalkozik a betegségemmel kapcsolatos új fejleményekkel.
7. The center I go to have my condition treated is well-located and easily accessible	7. A központ, ahol a betegségemet gondozzák jó elhelyezkedésű, könnyen megközelíthető.
8. The facilities at the hospital I go to treat my bowel disease are adequate and comfortable	8. A gondozásomat végző központ felszereltsége megfelelő és kényelmes.
9. Communication with the medical staff treating me is appropriate and fluid	9. A gondozásomat végző egészségügyi csapattal történő kommunikáció megfelelő és folyamatos.
10. I feel listened to and understood by the medical staff treating me when I explain my intestinal problems and the difficulties they cause me	10. Úgy érzem, hogy az engem ellátó egészségügyi dolgozók meghallgatják és megértik a bélproblémáimat és azokat a nehézségeket, amelyeket a betegségem okoz számomra.
11. I worry about the price I have to pay for the drugs prescribed for my bowel disease	11. Aggaszt a betegségemre felírt gyógyszerek ára.
12. For me, it is important that I always see the same medical team	12. Számomra fontos, hogy mindig ugyan azt a személyzetet lássam.
13. The staff take into account the consequences of my bowel disease treatment on my daily life	13. Az egészségügyi személyzet (pl.: orvos, nővér) figyelembe veszi a bélbetegségem kezelésének mindennapi életemre gyakorolt következményeit (pl.: rendszeres gyógyszerszedés, iskolai hiányzás, fáradékonyság).

14. I have been informed about how to contact with patients' associations for people with intestinal problems like mine	14. Tájékoztatást kaptam arról, hogyan tudok kapcsolatba lépni betegklubbokkal, ahová hozzám hasonló bélbetegséggel élők járnak.
15. Having a specialist nurse in the medical team treating me would help me with my bowel disease	15. Ha az engem ellátó egészségügyi csapatban lenne egy gyulladásos bélbetegség ellátására szakosodott nővér, az megkönnyítené számomra a betegségem ellátását.
16. The staff that look after me and the place I go for treatment motivate me to stick with the treatment for my illness	16. A betegségemet felügyelő egészségügyi személyzet és a hely, ahová kezelésekre járok, arra ösztönöz, hogy betartsam a javasolt orvosi utasításokat.
17. The center where they administer my medication has the necessary resources and facilities	17. A központ, ahol a kezeléseimet kapom, rendelkezik a szükséges erőforrásokkal és lehetőségekkel (pl.: vizsgálatok elérhetősége, képalkotó berendezések, személyzeti háttér).
18. At the hospital where I get treatment for my bowel disease, I can get information about my disease through brochures, information campaigns, etc	18. A kórházban, ahol a bélbetegségemmel kezelnek, tájékoztatást kaphatok a betegségemről ismeretterjesztő füzetek és kampányok formájában.
19. I can see the clinician when I have a flare-up	19. El tudok menni a kezelőorvosomhoz, amikor a betegségem újra fellángol.
20. Being able to talk with people who have the same or similar problems as me while I am receiving my medication, helps me to share questions and concerns related to my bowel disease	20. Kezelésem során olyan emberekkel beszélhetek, akik ugyanazokkal vagy hasonló problémákkal küzdenek, mint én és ez lehetővé teszi a betegségemmel kapcsolatos kérdések és aggodalmak megvitatását.
21. Visits can be scheduled on days and at times that least affect my daily activities (work, studies ...)	21. A rendszeres orvosi vizsgálatok olyan napokra és időpontokra időzíthetők, hogy azok a legkevésbé befolyásolják a mindennapi tevékenységemet (munka, iskolai tanulmányok...).
22. In the hospital, they treat me with sufficient intimacy and reserve	22. A kórházi ellátásom megfelelő és kellően bizalmas.
23. The time I have to wait before being seen at the visit is reasonable	23. A vizitek előtti várakozási idő elfogadható.
24. In the center I go to for treatment, I can be attended over the phone	24. Telefonon tudok időpontot foglalni a kórházba, ahová kezelésekre járok.
25. The bathrooms in the center are adequate and accessible	25. A kórházban található illemhelységek megfelelők és hozzáférhetők.
26. There is good coordination and communication between my medical team and other specialists and/or primary care	26. Az ellátásomat végző egészségügyi személyzet és más szakemberek, valamint az alapellátás (családorvos) között összehangolt együttműködés és kommunikáció zajlik.
27. I'm satisfied with the results of the treatment I receive	27. Meg vagyok elégedve a kezelésem során elért eredményekkel.
28. If any problems arise with the treatment I am receiving, my medical team resolve it quickly and effectively	28. Ha bármilyen probléma merül fel a kezelésemmel kapcsolatban, akkor orvosom és az egészségügyi csapat azt gyorsan és hatékonyan megoldja.
29. I feel safer if I get the treatment at the hospital than if I had to do it at home	29. Biztonságosabbnak érzem, ha a kórházban kapom meg a kezeléseket, mint otthon.
30. I understand the instructions I've been given about my medication	30. Megértem a gyógyszerelésemmel kapcsolatban kapott utasításokat.
31. I've been given adequate information about the side effects of my medication	31. Megfelelő tájékoztatást kaptam a gyógyszereim mellékhatásairól.

Supplementary Table S5 The mean and the Cronbach's α values in the CACHE questionnaires

Domains	mean values of patients' satisfaction (SD)		Cronbach's α	
	adolescent population	adult population	adolescent population	adult population
staff care	82.947 (±21.635)	76.506 (±26.069)	0.872	0.919
clinical care	83.826 (±21.294)	82.327 (±21.436)	0.704	0.806
centre facilities	78.247 (±24.762)	77.374 (±25.832)	0.639	0.774
patient information	60.382 (±33.762)	58.374 (±33.562)	0.550	0.685
accessibility	78.594 (±26.621)	70.375 (±28.866)	0.670	0.759
patient support	58.893 (±31.069)	60.773 (±29.566)	0.513	0.415

Supplementary Table S6 Correlation between total and subscores of the CACHE questionnaire and demographic data in the adolescent population

demographic variables	total score (p)	staff care items (p)	clinical care items (p)	center facilities items (p)	patient information items (p)	accessibility items (p)	patient support items (p)
gender	0.984	0.624	0.714	0.765	0.630	0.822	0.965
ethnicity	0.303	**0.029**	0.274	0.594	0.712	0.407	0.995
age at adaptation[1]	0.167	0.251	0.220	0.541	0.215	0.192	0.550
disease duration time[1]	0.903	0.482	0.856	0.434	0.579	0.805	0.950
disease type	0.052	**0.019**	0.236	0.581	0.069	0.083	0.531
previous intestinal surgery	0.425	0.482	0.551	0.372	0.705	0.139	0.503
number of medications	0.911	0.735	0.597	0.405	0.594	0.463	0.102
biological treatment	0.367	0.457	0.098	0.232	0.957	0.749	0.374
steroid treatment	0.754	0.909	0.826	0.856	0.231	0.469	0.199
azathioprine treatment	0.278	0.781	0.725	0.826	0.058	0.408	0.328
5-ASA treatment	0.743	0.699	0.927	0.529	0.472	0.649	0.350
comorbidities	0.235	0.305	0.124	0.479	0.175	0.212	0.068

[1]: results are given in correlation coefficients (ϱ)

Significant results are shown in bold.

Supplementary Table S7 Correlation between total and subscores of the CACHE questionnaires and demographic data in the adult population

demographic variables	total score (p)	staff care items (p)	clinical care items (p)	center facilities items (p)	patient information items (p)	accessibility items (p)	patient support items (p)
gender	**0.019**	**0.026**	0.062	**<0.001**	**0.027**	0.334	0.091
ethnicity	0.086	0.322	0.341	0.246	0.134	0.067	0.059
age at adaptation[1]	0.628	0.331	0.717	0.250	0.576	0.517	0.139
disease duration time[1]	0.178	0.26	**0.016**	0.977	0.243	0.115	0.314
disease type	**0.014**	**0.042**	**0.017**	0.330	0.08	**0.010**	0.170
previous intestinal surgery	0.184	0.361	0.479	0.786	0.177	0.072	0.277
number of medications	0.059	0.052	**0.002**	0.172	0.484	0.098	0.760
biological treatment	**0.005**	**0.006**	**0.006**	**0.003**	0.358	**0.032**	**0.001**
steroid treatment	0.214	0.365	0.181	0.147	0.357	0.125	0.763
azathioprine treatment	0.283	0.363	0.579	0.360	0.085	0.528	0.935
5-ASA treatment	0.087	0.138	0.544	0.068	0.086	0.067	0.128
comorbidities	0.750	0.253	0.497	0.403	0.973	0.823	0.288

[1]: results are given in correlation coefficients (ϱ)

Significant results are shown in bold.

Supplementary Table S8 Mean scores from the CACHE questionnaire for adolescents and adults

	Scores	Mean scores (SD) adolescents' population	adults' population
1	My doctor spends an appropriate amount of time listening to and answering my questions about my bowel disease	90.2 (±16.2)	81.3 (±23.7)
2	I have confidence in my doctor's judgment when managing and treating my bowel disease	91.6 (±16.6)	86.7 (±20.1)
3	understand the explanations given to me on my bowel disease, its treatment, and the side effects of treatment	85.1 (±17.8)	83.6 (±18.3)
4	I get advice and guidance about nutrition, daily activities, exercise, etc, which I have to follow because of my bowel disease	77.7 (±24.3)	69.6 (±29.0)
5	My doctor takes my opinion and preferences regarding treatment for my bowel disease into account	84.4 (±19.6)	80.1 (±22.8)
6	The medical personnel who look after me know my medical history and concern themselves with the evolution of my bowel disease	83.8 (±20.8)	75.6 (±28.3)
7	The center I go to have my condition treated is well-located and easily accessible	76.4 (±26.2)	83.6 (±21.0)
8	The facilities at the hospital I go to treat my bowel disease are adequate and comfortable	80.9 (±22.0)	79.8 (±23.5)
9	Communication with the medical staff treating me is appropriate and fluid	85.2 (±18.7)	77.8 (±26.4)
10	I feel listened to and understood by the medical staff treating me when I explain my intestinal problems and the difficulties they cause me	84.0 (±21.1)	80.1 (±22.9)
11	I worry about the price I have to pay for the drugs prescribed for my bowel disease	33.0 (±31.4)	39.6 (±30.6)
12	For me, it is important that I always see the same medical team	67.8 (±26.0)	80.0 (±21.4)
13	The staff take into account the consequences of my bowel disease treatment on my daily life	84.3 (±19.7)	77.8 (±22.6)
14	I have been informed about how to contact with patients' associations for people with intestinal problems like mine	51.3 (±34.0)	52.0 (±35.4)
15	Having a specialist nurse in the medical team treating me would help me with my bowel disease	62.0 (±29.1)	64.4 (±27.2)
16	The staff that look after me and the place I go for treatment motivate me to stick with the treatment for my illness	81.8 (±23.0)	80.2 (±22.1)
17	The center where they administer my medication has the necessary resources and facilities	86.6 (±19.4)	82.5 (±21.9)
18	At the hospital where I get treatment for my bowel disease, I can get information about my disease through brochures, information campaigns, etc	59.0 (±29.6)	61.3 (±33.0)
19	I can see the clinician when I have a flare-up	88.4 (±20.4)	77.6 (±25.6)
20	Being able to talk with people who have the same or similar problems as me while I am receiving my medication, helps me to share questions and concerns related to my bowel disease	52.1 (±31.1)	53.7 (±32.3)
21	Visits can be scheduled on days and at times that least affect my daily activities (work, studies ...)	67.1 (±30.1)	64.5 (±32.8)
22	In the hospital, they treat me with sufficient intimacy and reserve	87.0 (±20.7)	78.7 (±24.6)
23	The time I have to wait before being seen at the visit is reasonable	74.2 (±25.0)	66.9 (±26.3)
24	In the center I go to for treatment, I can be attended over the phone	84.7 (±20.2)	72.5 (±28.6)
25	The bathrooms in the center are adequate and accessible	69.0 (±27.5)	63.7 (±30.8)
26	There is good coordination and communication between my medical team and other specialists and/or primary care	72.5 (±27.2)	56.2 (±32.9)
27	I'm satisfied with the results of the treatment I receive	80.4 (±21.5)	74.2 (±25.2)
28	If any problems arise with the treatment I am receiving, my medical team resolve it quickly and effectively	84.4 (±18.9)	75.8 (±25.4)
29	I feel safer if I get the treatment at the hospital than if I had to do it at home	62.5 (±32.1)	64.2 (±27.8)
30	I understand the instructions I've been given about my medication	85.7 (±20.9)	88.6 (±15.5)
31	I've been given adequate information about the side effects of my medication	81.3 (±23.2)	69.2 (±30.0)
	Total score	76.2 (±12.4)	71.9 (±16.2)

Supplementary Figure S1 Steps of adaptation

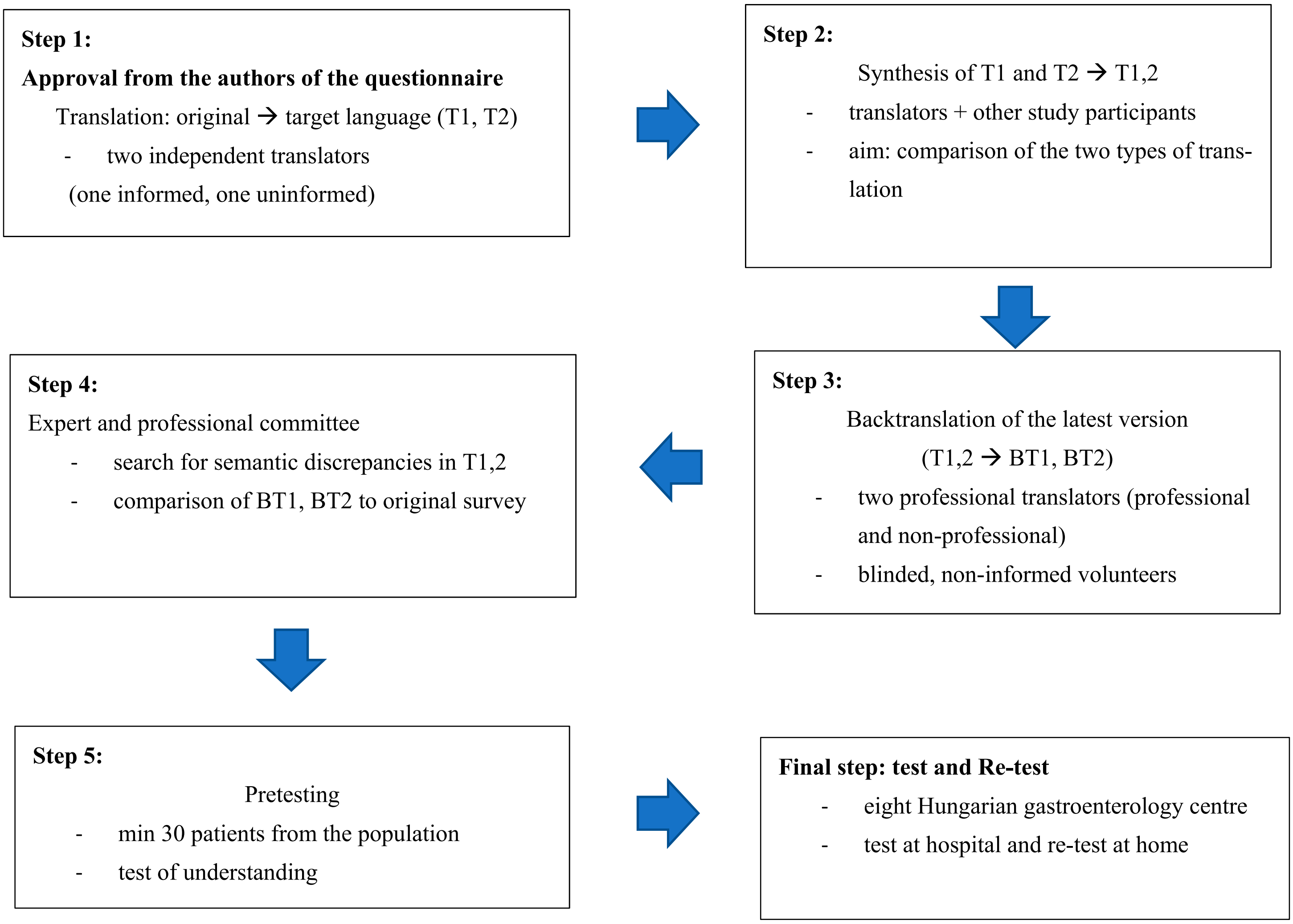

Supplement: Supplementary file 1 [file children-09-01143-s001.zip › children-1820511-supplementary.pdf]
